# LDCNN: A new arrhythmia detection technique with ECG signals using a linear deep convolutional neural network

**DOI:** 10.14814/phy2.16182

**Published:** 2024-09-01

**Authors:** Ali Bayani, Masoud Kargar

**Affiliations:** ^1^ Department of Computer Engineering, Tabriz Branch Islamic Azad University Tabriz Iran

**Keywords:** arrhythmia detection, cardiovascular health, convolutional neural network, deep learning, electrocardiogram

## Abstract

The electrocardiogram (ECG) is a fundamental and widely used tool for diagnosing cardiovascular diseases. It involves recording cardiac electrical signals using electrodes, which illustrate the functioning of cardiac muscles during contraction and relaxation phases. ECG is instrumental in identifying abnormal cardiac activity, heart attacks, and various cardiac conditions. Arrhythmia detection, a critical aspect of ECG analysis, entails accurately classifying heartbeats. However, ECG signal analysis demands a high level of expertise, introducing the possibility of human errors in interpretation. Hence, there is a clear need for robust automated detection techniques. Recently, numerous methods have emerged for arrhythmia detection from ECG signals. In our research, we developed a novel one‐dimensional deep neural network technique called linear deep convolutional neural network (LDCNN) to identify arrhythmias from ECG signals. We compare our suggested method with several state‐of‐the‐art algorithms for arrhythmia detection. We evaluate our methodology using benchmark datasets, including the PTB Diagnostic ECG and MIT‐BIH Arrhythmia databases. Our proposed method achieves high accuracy rates of 99.24% on the PTB Diagnostic ECG dataset and 99.38% on the MIT‐BIH Arrhythmia dataset.

## 1 INTRODUCTION

The human heart generates intricate electrical signals that provide vital insights into the cardiovascular system's functionality. These signals serve as diagnostic tools, offering crucial information about heart rate, rhythm, and potential abnormalities. Detecting cardiac anomalies can be challenging due to the complexity and diversity of cardiac diseases and the expertise required for their diagnosis. Electrocardiograms are crucial for rapidly assessing cardiac conditions and monitoring heart rhythm (Singh et al., 2019). Nevertheless, interpreting ECGs presents a substantial challenge due to the intricate nature of cardiac electrical activity and signal noise. In recent years, due to technological and architectural developments, deep learning has been very beneficial for analyzing and processing these signals (Hu et al., 2022).

**FIGURE 1:**
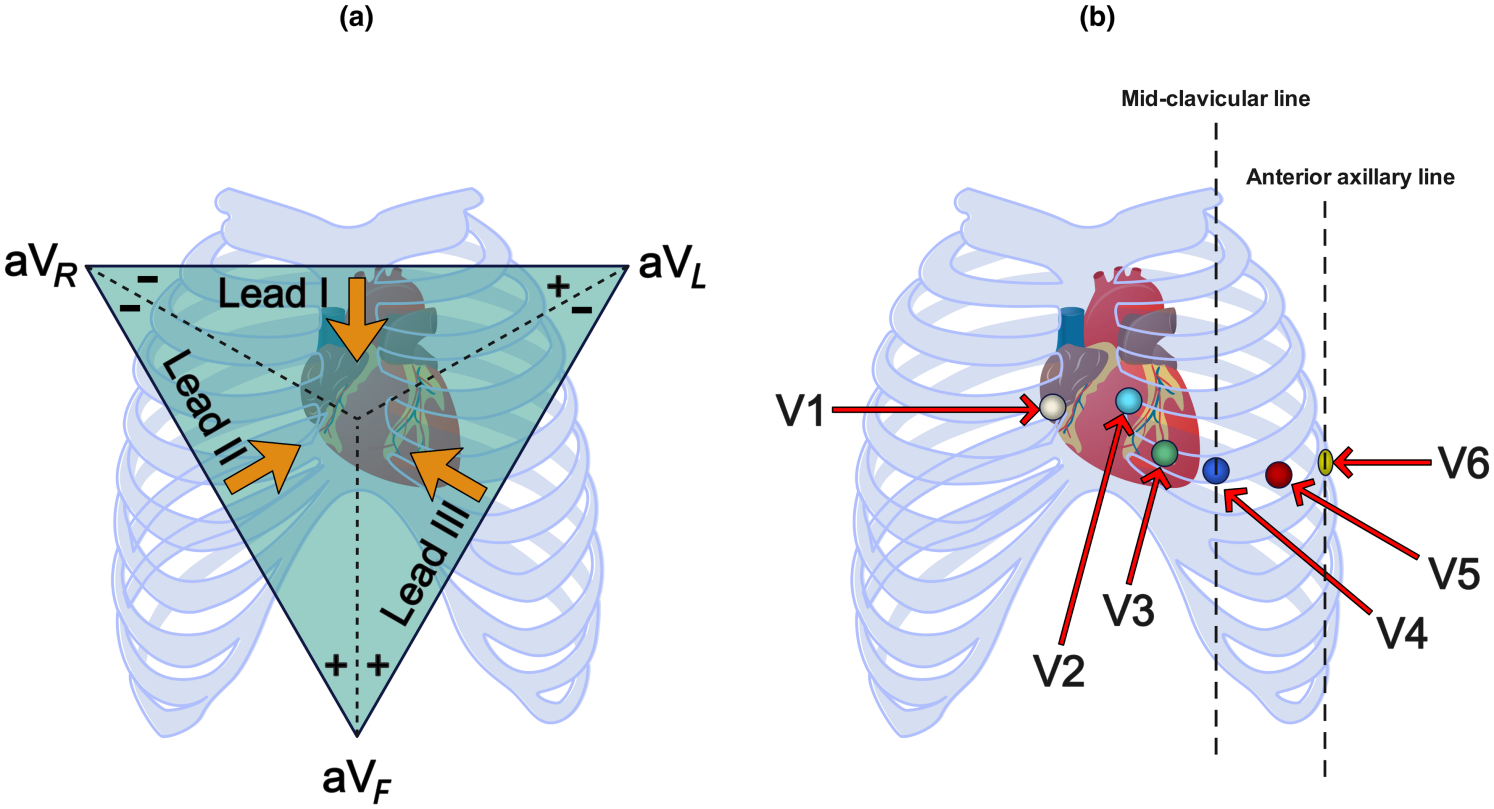
Types of ECG leads, including (a) the three primary leads and (b) the nine unipolar leads.

Cardiac signals hold potential for the diagnosis and investigation of heart diseases and encompass various types, including ECGs (Berkaya et al., 2018), photoplethysmograms (PPGs) (González et al., 2023), arterial blood pressure (ABP) (Arvanaghi et al., 2017), cardiac output (CO) (Mehta & Arora, 2014), heart rate variability (HRV) (Brockmann & Hunt, 2023), and more. These signals vary in signal type, frequency domain, voltage, and measurement method, commonly utilized in the analysis of heart diseases. ECG is a prevalent noninvasive method for measuring heart rate, examining heart rhythm, and timely detecting cardiac irregularities (Alberdi et al., 2016; Merone et al., 2017). ECG records the heart's electrical signals during blood circulation throughout the body, producing insights into cardiac activity (Burgess, 2022). This signal comprises distinct waveform shapes (P, QRS, and T), with each shape representing specific cardiac activities. Various heart diseases manifest differently in ECG waveform shapes. The ECG signal comprises several leads, as illustrated in Figure 1 (a) the three primary leads (I, II, and III) and (b) the nine unipolar leads (V1–V6, aVR, aVL, and aVF). However, ECG signals face challenges such as noise, power line interference, baseline drift, and more (Friesen et al., 1990). ECG signal analysis requires extensive expertise due to its complexity and imperfections. Therefore, human error in ECG interpretation underscores the need for an efficient and accurate automated ECG detection system (Ribeiro et al., 2020).

Cardiac diseases encompass a range of cardiovascular conditions resulting from dysfunction of the heart and blood vessels. Among them, arrhythmia holds particular significance due to its impact on heart rhythm, rate, and regularity (Hu et al., 2022; Singh et al., 2019). Arrhythmias have various types classified based on their origin within the heart. Examples include ventricular arrhythmia, characterized by premature ventricular contractions (PVC), ventricular tachycardia (VT), and ventricular fibrillation (VF) (Mazidi et al., 2020). Ventricular premature contractions involve premature heartbeats originating from the Purkinje fibers rather than the sinoatrial node (Hurley et al., 2023). Ventricular tachycardia is marked by irregular and rapid heartbeats, with monomorphic ventricular tachycardia being the most common type (Zhang et al., 1999). Ventricular fibrillation (Wang et al., 2007) results in completely irregular and swift heartbeats in the ventricles, causing erratic contractions and trembling of the heart (Tseng & Tseng, 2020). Additionally, Torsades de Pointes, a form of polymorphic ventricular tachycardia characterized by a twisting pattern on the electrocardiogram, poses a distinct risk, especially in the context of prolonged QT intervals (Leenhardt et al., 2012). Supraventricular tachycardia (SVT) entails a faster‐than‐normal heart rate in the atria, with several variations (Grubb et al., 2020). Bradyarrhythmia manifests as a significantly slow heart rate, often associated with heart failure or sinus node dysfunction (Sidhu & Marine, 2020). We can categorize arrhythmias as either morphological, characterized by irregularities in the shape or structure of the heart's electrical signals, or rhythmic, caused by sets of irregular heartbeats. Accurate and timely diagnosis of cardiac arrhythmias is crucial, as these irregularities can manifest as disruptions in rhythm, alterations in conduction, or changes in repolarization patterns that may suggest underlying disease or altered physiological states.

Deep learning is a formidable approach within the machine learning domain, leveraging deep neural networks to extract high‐level features from data. In the context of signal processing, the application of deep learning, particularly for the analysis of ECG signals, has proven to be highly effective and practical (Murat et al., 2020). Deep neural networks can identify and utilize significant latent features within ECG signals to classify and diagnose cardiac diseases (Liu et al., 2021).

In the field of arrhythmia detection, multiple methods for analyzing ECG signals exist, and we can categorize them into two main groups: non‐deep learning‐based methods, which include traditional machine learning algorithms or signal processing techniques, and deep learning‐based methods.

### 1.1 Non‐deep learning‐based methods

Asl et al. (2008). proposed a classification algorithm for cardiac arrhythmias using heart rate variability (HRV) signals. They employed the MIT‐BIH Arrhythmia Database to diagnose six different types of cardiac arrhythmias. Their approach improved the classification metrics by selecting optimal features and achieved accuracies of 98.94%, 98.96%, 98.53%, 98.51%, 100%, and 100% for six arrhythmia classes (2008). Hadj Slimane et al. (2010). introduced a novel algorithm for complex QRS detection using Empirical Mode Decomposition in ECG signals. This algorithm employed low‐pass and high‐pass filters, empirical mode decomposition of signals, and a nonlinear transformation. They evaluated their technique on the MIT–BIH dataset and achieved accuracy results of 95.58% accuracy, 99.84% sensitivity, and 99.92% specificity (2010). Kutlu et al. (2012) indicated an automated heartbeat detection method utilizing higher order statistics of wavelet packet decomposition coefficients. They classified using the K‐Nearest Neighbors algorithm. The experiments were performed on the MIT‐BIH dataset, resulting in an average sensitivity of 90%, average selectivity of 92%, and average specificity of 98% (2012). Raj et al. (2016) proposed a method for classifying cardiac arrhythmia beats using discrete orthogonal Stockwell transform (DOST) and Support Vector Machine (SVM) with Particle Swarm Optimization (PSO) tuning. The method was validated on the MIT‐BIH dataset, achieving overall accuracies of 99.18% for 16 classes and 89.10% for five classes. PSO improved classification accuracy, with symmetry features contributing significantly to this improvement (2016). Sahoo et al. (2017). introduced an improved algorithm for detecting complex QRS features using the Multiresolution Wavelet Transform for classifying four types of ECG beats, including arrhythmias. Their approach's performance was evaluated for accuracy, sensitivity, and specificity on 48 ECG signals from the MIT‐BIH dataset, reaching average accuracies of 96.67% and 98.39% in ANN (Artificial Neural Network) and SVM (Support Vector Machine), respectively (2017).

### 1.2 Deep learning‐based methods

Abdalla et al. (2019) in their research, focused on the Classification of ECG arrhythmias using nonlinear and nonstationary decomposition methods. They used the full ensemble empirical mode decomposition with adaptive noise (CEEMDAN) approach to extract the intrinsic mode functions (IMFs). They used the four parameters of these functions to construct the feature vector. Consequently, they used the artificial neural network to apply the feature vector and classify five different types of arrhythmia heartbeats using the MIT‐BIH database. The results showed that the CEEMDAN and ANN approach performed very well, with 99.9% accuracy (2019). Zairi et al. (2020) introduced an FPGA‐based arrhythmia detection system utilizing an artificial neural network for real‐time cardiac disease detection. This method involved Wavelet Transform for feature extraction, a Multilayer Perceptron (MLP) for classification and arrhythmia detection, and decision‐making based on the ANN output. The evaluation, conducted using the MIT‐BIH dataset, resulted in an average sensitivity of 98.33% and an accuracy of 98.2% (2020). Gupta et al. (2020) utilized three techniques: a novel fractional wavelet transform (FrWT), Yule‐Walker Autoregressive Modeling, and Principal Component Analysis (PCA) for denoising, feature extraction, and dimensionality reduction. They evaluated their approach on the MIT‐BIH dataset, achieving an accuracy of 99.94% and 99.89% for the Real‐time ECG and MIT‐BIH databases, respectively (2020). Table 1 presents the advantages and disadvantages of each of the above approaches.

**TABLE 1:** Summary of previous works and their advantages and disadvantages.

Year	Author	Title	Algorithm	Dataset	Advantages	Disadvantages
2008	Babak Mohammadzadeh Asl, Seyed Kamaledin Setarehdan and Maryam Mohebbi	Support vector machine‐based arrhythmia classification using reduced features of heart rate variability signal	Algorithm based on Generalized Discriminant Analysis (GDA) Feature Reduction and Support Vector Machine (SVM) Classifier	MIT‐BIH	1. Diagnosis with high accuracy. 2. Reduction of processing time and possibly creating an online arrhythmia detection system. 3. Reducing the number of features increases the accuracy of decision‐making	1. Inability to recognize arrhythmias such as left bundle branch block and right bundle branch block. 2. Dependence on specific HRV signals. 3. Loss of important information in the ECG signal
2010	Zine‐Eddine Hadj Slimane and Amine Naït‐Ali	QRS complex detection using Empirical Mode Decomposition	Empirical Mode Decomposition Algorithm	MIT‐BIH	1. Multiple steps with high accuracy. 2. Significant improvement in QRS complex detection	1. Computational time complexity leads to slowness in processing. 2. Dependence on specific MIT‐BIH datasets. 3. Complexity of algorithm implementation
2012	Yakup Kutlu and Damla Kuntalp	Feature extraction for ECG heartbeats using higher order statistics of WPD coefficients	1. Higher order statistics (HOS) of wavelet packet decomposition (WPD) coefficients 2. KNN	MIT‐BIH	1. High accuracy in heart rate detection. 2. WPD noise removal has a higher frequency resolution. 3. Strong performance in classification with KNN	1. Computational complexity in using higher order statistics calculation. 2. Poor performance in diagnosing other types of cardiac disorders. 3. Low detection accuracy in unusual conditions. 4. Reduction of KNN performance in large and complex data
2016	Sandeep Raj, Kailash Chandra Ray, and Om Shankar	Cardiac arrhythmia beat classification using DOST and PSO‐tuned SVM	1. Discrete orthogonal Stockwell transform (DOST) 2. Particle swarm optimization (PSO) 3. The support vector machine (SVM) classifier	MIT‐BIH	1. Improving the accuracy of diagnosis and reducing mistakes. 2. Better diagnosis in the face of dynamic changes	1. Computational complexity and the need for high processing power. 2. Increasing execution time in parameter optimization with PSO
2017	Santanu Sahoo, Bhupen Kanungo, Suresh Behera and Sukanta Sabut	Multiresolution wavelet transform‐based feature extraction and ECG classification to detect cardiac abnormalities	1. Multiresolution wavelet transform 2. Neural network (NN) 3. Support vector machines (SVM) classifiers	MIT‐BIH	1. High accuracy in diagnosis and classification. 2. Noise removal	Increase computational complexity using wavelet transform
2019	Fakheraldin Y. O. Abdalla, Longwen Wu, Hikmat Ullah, Guanghui Ren, Alam Noor and Yaqin Zhao	ECG arrhythmia classification using artificial intelligence and nonlinear and nonstationary decomposition	Nonlinearity and nonstationary decomposition methods using complete ensemble empirical mode decomposition with adaptive noise (CEEMDAN)	MIT‐BIH	1. Extracting special features to diagnose cardiac irregularities. 2. High accuracy in diagnosis and classification	It has computational complexity due to the use of nonlinear analysis methods and the extraction of particular features
2020	Hadjer Zairi, Malika Kedir Talha, Karim Meddah and Saliha Ould Slimane	FPGA‐based system for artificial neural network arrhythmia classification	1. Field programmable gate array (FPGA) 2. Wavelet transform 3. Multilayer perception (MLP)	MIT‐BIH	1. High accuracy and generalizability. 2. Minimizing features and reducing energy consumption. 3. Fast and real‐time implementation 4. Long‐term and interactive monitoring. 5. Low error rate in data classification	1. Complexity of FPGA implementation. 2. Resource limitations. 3. Using an FPGA chip is expensive and time‐consuming. 4. Limitation in the diagnosis of some diseases. 5. In some cases, there is a decrease in classification accuracy and errors in pattern recognition
2020	Varun Gupta and Monika Mittal	Arrhythmia detection in ECG signal Using fractional wavelet transform with principal component analysis	1. Fractional wavelet transform (FrWT) 2. Yule‐Walker autoregressive modeling (YWARM) 3. Principal component analysis (PCA)	MIT‐BIH	1. Noise cleaning. 2. Improve detection accuracy	1. More time is needed for processing due to the higher computational complexity of combining methods. 2. Due to the use of variance estimation with PCA, there is a possibility of less accuracy in diagnosing some cardiac irregularities. 3. Optimizing the method's performance requires better configuration and parameter setting

While traditional machine learning techniques offer advantages, they often encounter challenges and are prone to errors in feature extraction, classification, and interpretation of ECG signals for heart disease detection. These conventional methods come with challenges such as complexity, the vast size of datasets, and ambiguity in detection, requiring parameter optimization and tuning. On the contrary, deep convolutional neural networks, among other deep learning approaches, leverage multilayer neural networks and automation to automatically extract features. They address some of the issues of traditional methods with lower complexity.

In our research, we develop a novel technique based on a one‐dimensional deep convolutional neural network. This technique aims to extract vital information from cardiac signals across various frequencies and represent them as vectors instead of images, reducing errors. Moreover, we have significantly improved this algorithm's complexity and execution time. For this reason, we have introduced it as a one‐dimensional linear deep convolutional neural network (LDCNN).

- Research Question 1: How effectively does our proposed linear deep convolutional neural network (LDCNN) model diagnose cardiac diseases across diverse datasets?
- Research Question 2: What is the comparative performance of our proposed LDCNN model in diagnosing cardiac diseases, and how does it specifically outperform traditional machine learning methods across different datasets?
- Research Question 3: How does the diagnostic accuracy of the LDCNN model vary across different arrhythmia classes, and what insights can be gained from the precision, recall, and F1‐score metrics for each class across diverse datasets?
- Research Question 4: What trends are observed in the training and testing accuracy, as well as loss, during the training epochs of the LDCNN model, and how do these trends contribute to the model's overall effectiveness in diagnosing cardiac diseases across various datasets?

## 2 METHODS

### 2.1 Motivation

With the continuous evolution of medical diagnostics, the precise detection and classification of arrhythmias have gained paramount significance. Given the increasing prevalence of cardiovascular diseases, the necessity for accurate and efficient arrhythmia analysis techniques has become more critical than ever before. Among these techniques, the utilization of convolutional neural networks has demonstrated better outcomes. Traditional methods have shown limitations in handling the complex characteristics of ECG signals, often needing higher performance accuracy. Hence, our proposed deep linear convolutional neural network provides a comprehensive solution that overcomes the existing challenges. This paper addresses the unexplored potential of this technique and paves the way for innovative advances in enhancing heart health diagnostics.

### 2.2 Datasets

We review the datasets utilized for simulating our proposed method, leveraging two widely employed benchmark datasets in contemporary research: the PTB Diagnostic ECG (Bousseljot et al., 1995; Kachuee et al., 2018) and MIT‐BIH Arrhythmia (Moody & Mark, 2001) datasets.

#### 2.2.1 PTB Diagnostic ECG dataset

PTB Diagnostic ECG includes a set of samples that are used to diagnose cardiovascular abnormalities. This dataset contains 549 records from 290 people. Each record contains 15 signals measured simultaneously, and these signals consist of 12 leads. This dataset includes two classes, normal and non‐normal. Table 2 contains the different types of heartbeats available in the PTB Diagnostic ECG dataset. Table 3 contains the heart rate types used in this article for this dataset and contains information about each one.

**TABLE 2:** Various primary categories of heartbeats are contained in the PTB diagnostic ECG dataset.

Beat description	Heartbeats
Normal beat, healthy controls	Normal beat
Cardiomyopathy/heart failure, myocarditis, myocardial infarction, miscellaneous, bundle branch block, dysrhythmia, valvular heart disease, and myocardial hypertrophy	Abnormal beat

**TABLE 3:** Types of heartbeats we used in the PTB diagnostic ECG dataset and overview of its beat annotations.

Heartbeats	Classes	Beat description	Frequency (%)	Count
Normal	Normal	Normal beat, healthy controls	27.81	4046
Abnormal	Abnormal	Cardiomyopathy/heart failure, myocarditis, and myocardial infarction Miscellaneous, bundle branch block, dysrhythmia, valvular heart disease, and myocardial hypertrophy	72.19	10,506

#### 2.2.2 MIT‐BIH Arrhythmia dataset

This is a standard dataset for the evaluation of various arrhythmia diseases. The dataset includes 48 dual‐channel ECG recordings, each lasting 30 min, collected from 47 patients over 4 years. It comprises five categories: normal beat, ventricular premature contraction, supraventricular premature beat, combined ventricular and normal beat, and unclassifiable beat. For this study, we selected a subset of these arrhythmias based on their clinical relevance and prevalence in real‐world scenarios. The selection process aimed to focus on representative arrhythmias to ensure a robust evaluation of our proposed methodology. Refer to Table 4 for the detailed breakdown of heartbeats in the MIT‐BIH Arrhythmia dataset. Table 5 provides an overview of the subset of heartbeats used in this article, along with their respective annotations, frequencies, and class labels.

**TABLE 4:** Various primary categories of heartbeats are contained in the MIT‐BIH Arrhythmia dataset.

Beat description	Heartbeats
Normal beat, atrial escape beat, nodal (junctional) escape beat, right bundle branch block beat, and left bundle branch block beat	Normal beat
Ventricular escape beat and premature ventricular contraction	Premature ventricular contraction
Supraventricular premature beat, atrial premature beat, nodal (junctional) premature beat, and aberrated atrial premature beat	Supraventricular premature beat
Fusion of normal and ventricular beat	Fusion beat
Fusion of normal and paced beat, and unclassifiable beat	Unclassifiable beat

**TABLE 5:** Types of heartbeats we used in the MIT‐BIH Arrhythmia dataset and overview of its beat annotations.

Heartbeats	Classes	Beat description	Frequency (%)	Count
Normal	N	Normal beat	75.0	75,011
	L	Left bundle branch block beat	8.07	8071
	R	Right bundle branch block beat	7.25	7255
Supraventricular	A	Atrial premature beat	7.13	7129
Ventricular	V	Premature ventricular contraction	2.55	2546

### 2.3 Proposed method

**FIGURE 2:**
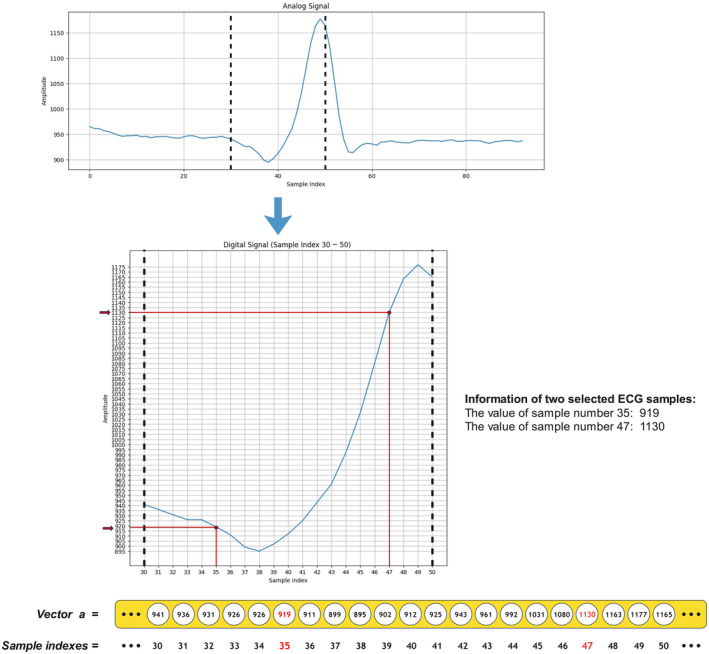
Visualization of an example signal in analog and digital forms, along with the vectorization process.

ECG signals are acquired and recorded in digital format, utilizing Analog‐to‐digital converters (ADCs) during the data acquisition process, subsequently forming the basis for the creation of datasets like MIT‐BIH and PTB. Advanced digital signal processing techniques are then applied for preprocessing and data conditioning, enabling the extraction of high‐level features by a deep convolutional neural network (DCNN). In Figure 2, we visually represent an example of analog signals from the MIT‐BIH Arrhythmia dataset using the Matplotlib library to illustrate how we use the data in our proposed model. The horizontal axis depicts the index of each sample, and the vertical axis represents the corresponding voltage values. We select a specific range of observations from index 30 to 50, transforming them into a vector named “a.” Additionally, two random samples from this range are chosen, and their values are mentioned. This selected range is transformed into a vector called “a” and displayed. This reshaping and conversion into vectors, as detailed in phase (v) of our methodology, are crucial steps in preparing the data for subsequent processing. These vectors serve as inputs to the model.

**FIGURE 3:**
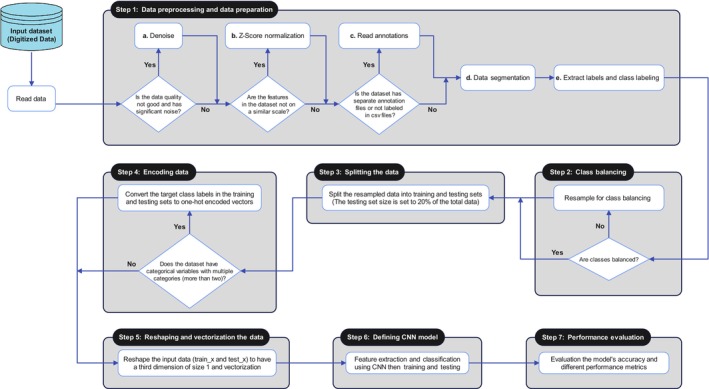
The general schema of our proposed method for arrhythmia detection from ECG signals.

Our proposed methodology comprises seven distinct phases: (i) data preprocessing and preparation, (ii) class balancing, (iii) data separation, (iv) data encoding, (v) data reshaping and vectorization, (vi) high‐level feature extraction utilizing LDCNN and classification, and (vii) model testing and evaluation. Figure 3 illustrates the general framework for ECG signal arrhythmia detection. In the following, we have discussed each of these phases.

#### 2.3.1 Data preprocessing and preparation

After converting the signals into digital form, we propose this process, which consists of five steps, to increase the accuracy and reliability of the classification process.

##### Denoising ECG signals

**FIGURE 4:**
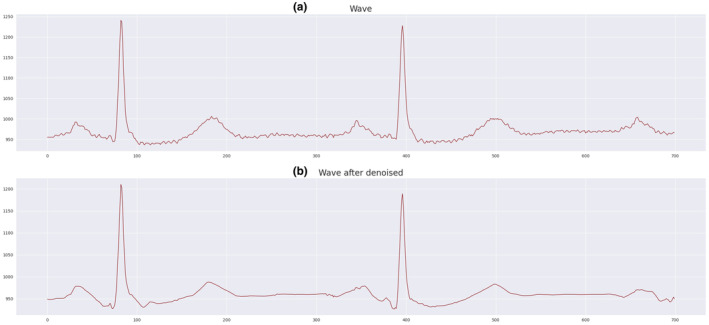
ECG signal (a) before and (b) after denoising.

ECG signals may contain errors due to noise, including interference from power lines. Such interference can reduce the accuracy of the analysis and lead to errors in diagnosing arrhythmia. Denoising is an approach to deal with these problems and increase the accuracy of ECG signal analysis. The Wavelet Thresholding method is a noise removal method that effectively separates the unwanted signal components from the desired signal and protects the key characteristics of the signal. This method is used if there is significant noise in the data. For example, this technique has been applied to the MIT‐BIH Arrhythmia dataset. Figure 4 shows instances of ECG signals (a) before and (b) after applying the denoising method.

##### ECG signal normalization

**FIGURE 5:**
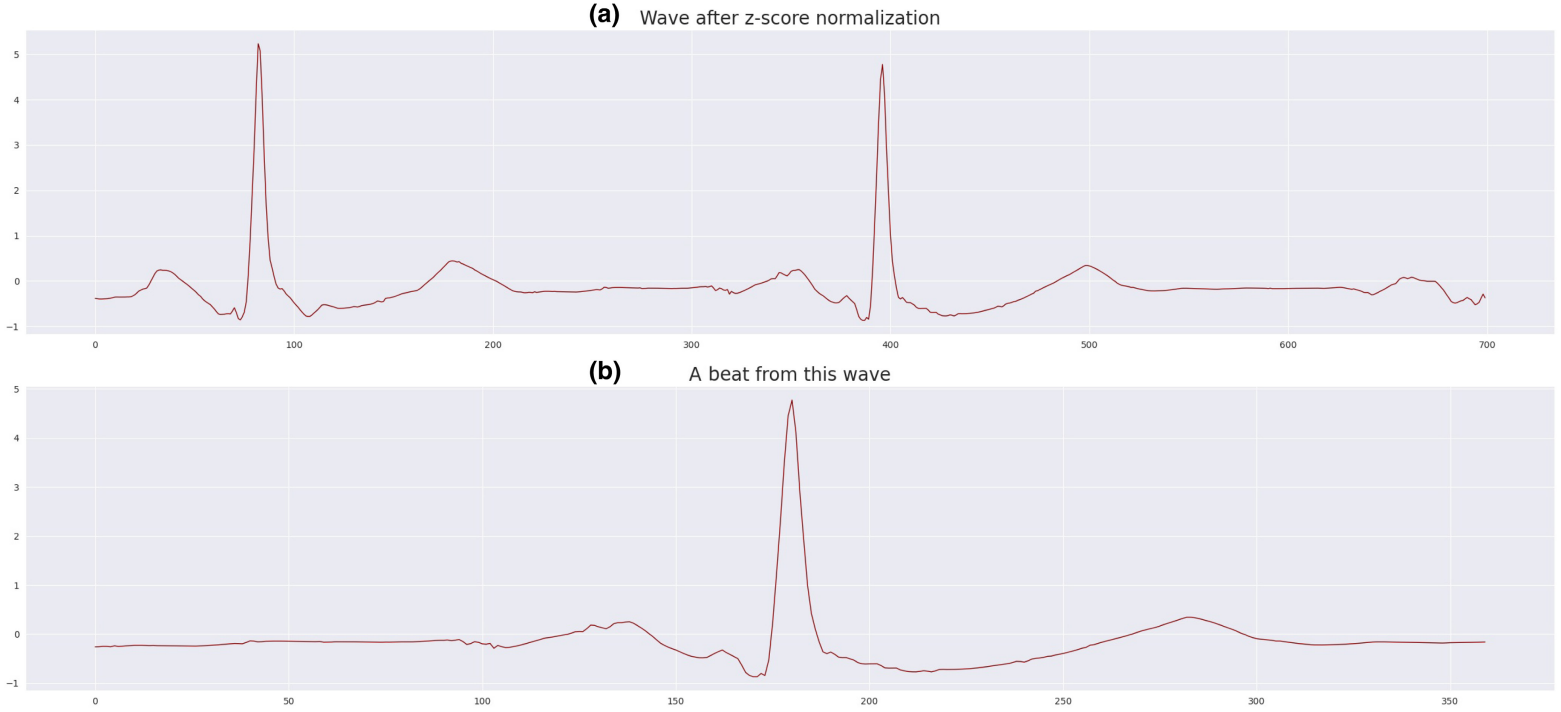
(a) ECG signal after normalization and (b) segmented ECG signal or beat from this wave.

Normalizing the data of ECG signals can significantly improve the accuracy of ECG signal analysis. Through normalization, we can transform the signal data onto a common scale and mitigate the effects of scale variations between different units. If the dataset features are not in a common scale, we have employed the z‐score technique for signal normalization. First, we calculate the data's mean and then compute the standard deviation. Then, using the *z*‐score method, we transform each point of the signal in a way that the mean becomes zero and the standard deviation becomes one. This process aligns the data within a specific range, making them comparable. Figure 5 (a) illustrates an example of ECG signals after applying the normalization method to the MIT‐BIH Arrhythmia dataset.

##### Reading annotations files

Reading annotations refers to the process of extracting information related to each heartbeat in a signal. Annotations typically provide additional information about events or specific features in the signal, such as the positions of R‐peaks (the highest point of the QRS complex in an ECG signal) and the corresponding arrhythmia class (such as normal beat, premature ventricular contraction). They contain information about the timing or location of important events in the signal and are often used for labeling signal data for further analysis or classification tasks. Due to the annotation files in the MIT‐BIH Arrhythmia dataset, we utilize this process. By reading annotation files, we can extract information related to each sample and utilize it in subsequent phases. This information is crucial for tasks like heartbeat classification and analysis, enabling us to understand the characteristics of each beat and make informed decisions based on the extracted features and annotations.

##### ECG signal segmentation

After completing the previous steps, the focus shifts to the vital components of the signal waves. In this context, segmentation is performed to extract only the necessary segments for model training. It is crucial to emphasize that a comprehensive view of the entire conduction cycle, including the P wave in PQRST, is essential for accurate delineation of specific heart blocks like SA or AV block, and for distinguishing between variations within each. Additionally, without consideration of the complete conduction cycle, differentiation between premature atrial contractions (PACs) and premature ventricular contractions (PVCs), as well as discerning non‐conducted PACs from AV block, can be challenging. In this phase, we utilize a technique called fixed‐size window segmentation and specifically extract the R‐peak from the ECG signals. The R‐peak, representing the peak of the QRS complex in the ECG signal, offers crucial information about heart rate and cardiac abnormalities. It is the distance from the beginning of the QRS complex to the maximum point of the R wave. The fixed‐size window segmentation involves extracting sections (windows) with a predefined and fixed length from the ECG signal, enhancing clarity and focus. Figure 5 (b) illustrates the segmentation of the ECG signal.

##### Class labeling

In this step, labels are assigned to the segmented ECG signal data based on the arrhythmia classes associated with each heartbeat. This process is necessary to train a machine learning model to accurately classify different types of arrhythmias. To obtain arrhythmia class information, we use annotations associated with ECG signals. Annotations provide details about the type of each heartbeat event. For example, they can represent classes such as A (atrial premature), R (right bundle branch block), L (left bundle branch block), V (premature ventricular contraction), N (normal), or abnormal. Tables 3 and 5 contain information about the classes within each dataset.

#### 2.3.2 Class balancing

**FIGURE 6:**
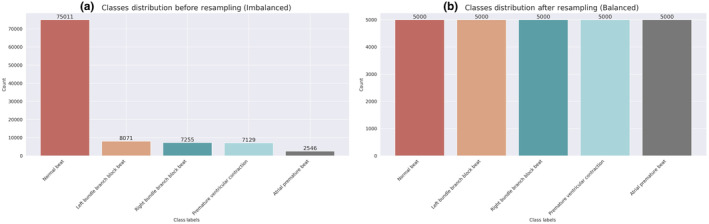
Resampling for balancing the classes of the MIT‐BIH Arrhythmia dataset: (a) class distribution before and (b) after resampling.

Normally, the uneven distribution in the number of samples of different classes of a dataset leads to problems in the field of ECG signal analysis. To solve these challenges and improve the efficiency of machine learning models, we have used the resampling technique. This technique involves generating additional samples from the minority class to achieve a more balanced distribution of samples among the different classes. Figure 6 illustrates the number of samples in the MIT‐BIH Arrhythmia dataset, with (a) showing the class distribution before and (b) showing the distribution after resampling. Additional details are provided in Table 6.

**TABLE 6:** The count of samples from distinct classes in the MIT‐BIH Arrhythmia dataset both before and after the resampling process.

Classes	Before resampling	After resampling
Premature ventricular contraction	2546	5000
Atrial premature beat	7129	5000
Right bundle branch block beat	7255	5000
Left bundle branch block beat	8071	5000
Normal beat	75,011	5000

#### 2.3.3 Data splitting

The data segmentation strategy is a significant step in preparing datasets for training and testing machine learning models. After resampling, we separated the data into two sets for training and testing, where the testing set contains 20% of the total data. This is done to evaluate the performance of the model and ensure that the model works correctly on new data.

#### 2.3.4 Data encoding

Encoding techniques in the field of machine learning refer to the process of converting categorical data into a numerical format that can be easily processed by machine learning algorithms. Categorical data includes labels or categories that do not have a natural numeric representation. Machine learning algorithms usually require numerical inputs, which is why encoding is essential. We have used the One‐Hot Encoding method in this section. In this method, we convert categorical data into binary vectors. If there are two categories for a categorical variable, it is often coded as the numbers zero and one. However, if suppose the categorical variable has more than two categories in a dataset, in that case, we usually treat each category as a binary feature and then convert these binary features to numeric values. According to the MIT‐BIH Arrhythmia dataset, it is assumed that there is a variable called arrhythmia in this dataset, whose values are “Normal beat,” “Right bundle branch block beat,” “Left bundle branch block beat,” “Atrial premature beat,” and “Premature ventricular contraction.” As shown in Table 7, all the values of this variable are converted into five separate columns with five samples. Now, for the first sample, which is “Normal beat,” the number 1 is entered in the Normal beat column and zero in the rest of the columns. For the second sample, which is “Right bundle branch block beat,” in the columns “Normal beat,” “Left bundle branch block beat,” “Atrial premature beat,” and “Premature ventricular contraction,” the value is zero, and, in the column, “Right bundle branch block beat,” “The value one is entered. It is done in the same way for other samples.”

**TABLE 7:** Encoding classes for the MIT‐BIH Arrhythmia dataset.

Arrhythmia	Normal beat	Right bundle branch block beat	Left bundle branch block beat	Atrial premature beat	Premature ventricular contraction
Normal beat	1	0	0	0	0
Right bundle branch block beat	0	1	0	0	0
Left bundle branch block beat	0	0	1	0	0
Atrial premature beat	0	0	0	1	0
Premature ventricular contraction	0	0	0	0	1

#### 2.3.5 Data reshaping and vectorization

The data are represented as a tensor with the shape (rows, columns, layers, and number of samples). We have transformed the input data to have a third dimension of size one. In our research and code implementation, the process of data reshaping plays an essential role in preparing our datasets for neural network training. This process involves transforming the input data arrays to conform to the specific structure required by the neural network architecture and is very important when working with CNNs, especially when handling one‐dimensional signals such as ECG data. This step is integral to the preprocessing step and serves as an effective strategy to handle class‐specific irregularities in diverse datasets, thereby increasing the flexibility and accuracy of ECG signal analysis. After doing this, we have applied the vectorization process. A process in which data, often represented as an array or matrix, is converted to a one‐dimensional vector. This transformation facilitates ease of processing for algorithms and operations designed to work with linear data.

#### 2.3.6 Definition of LDCNN

**FIGURE 7:**
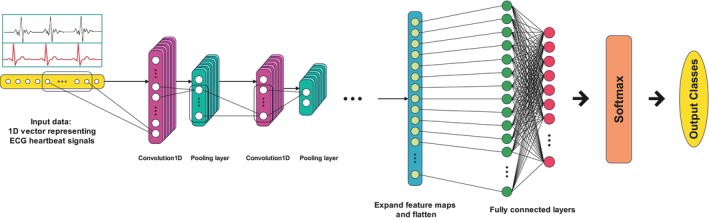
The architecture of our proposed LDCNN model.

Our proposed technique, known as linear deep convolutional neural network (LDCNN), consists of a linear one‐dimensional deep convolutional neural network. In this technique, one‐dimensional linear convolution is used deeply, which means using several layers. Using this method, we can extract high‐level features from the ECG signal and recognize patterns. The use of convolution in this technique helps us to extract important features due to the unknown location of arrhythmia and the absence of a specific time to observe cardiac arrhythmia. The data are one‐dimensional and in the form of an input signal. But when these signals are sampled, they become a vector form of a one‐dimensional tensor. Therefore, by using linear convolution, we can extract patterns from these vectors and recognize the moment of the event based on the previous steps. For this reason, this technique is known as LDCNN, and its use increases the accuracy. The general structure of our proposed model is shown in Figure 7.

In the LDCNN architecture for PTB Diagnostic ECG and MIT‐BIH Arrhythmia dataset, eight and five convolution layers are considered, respectively, and each of these layers is associated with a pooling layer. After the flattening layer, a dropout layer is subsequently applied to generate the final feature vector. Tables 8 and 9 contain information about each architecture and hyperparameters of each.

**TABLE 8:** The diverse hyperparameters used in configuring our LDCNN model for the MIT‐BIH Arrhythmia dataset.

Layer name	Filters	Kernel size	Activation function	Input shape	Output shape	Dropout rate	Pool size (Avg)	Padding	Stride
Input layer	‐	‐	‐	1 × 360	‐	‐	‐	‐	‐
Conv1D layer 1	16	13	ReLU	1 × 360	16 × 360	‐	‐	Same	1
AvgPool1D	‐	‐	‐	16 × 360	179 × 16	‐	3	‐	2
Conv1D layer 2	32	15	ReLU	179 × 16	32 × 179	‐	‐	Same	1
AvgPool1D	‐	‐	‐	32 × 179	32 × 89	‐	3	‐	2
Conv1D layer 3	64	17	ReLU	32 × 89	64 × 89	‐	‐	Same	1
AvgPool1D	‐	‐	‐	64 × 89	64 × 44	‐	3	‐	2
Conv1D layer 4	128	19	ReLU	64 × 44	128 × 44	‐	‐	Same	1
AvgPool1D	‐	‐	‐	128 × 44	128 × 21	‐	3	‐	2
Conv1D layer 5	256	21	ReLU	128 × 21	256 × 21	‐	‐	Same	1
AvgPool1D	‐	‐	‐	256 × 21	256 × 10	‐	3	‐	2
Flatten layer	‐	‐	‐	256 × 10	2560	‐	‐	‐	‐
Dropout layer	‐	‐	‐	2560	2560	0.5	‐	‐	‐
Dense layer 1	35	‐	‐	2560	35	‐	‐	‐	‐
Dense layer 2	5	‐	‐	35	5	‐	‐	‐	‐
Output layer	‐	‐	Softmax	5	5	‐	‐	‐	‐

**TABLE 9:** The diverse hyperparameters used in configuring our LDCNN model for the PTB diagnostic ECG dataset.

Layer name	Filters	Kernel size	Activation function	Input shape	Output shape	Dropout rate	Pool size (max)	Padding	Stride
Input layer	‐	‐	‐	1 × 187	‐	‐	‐	‐	‐
Conv1D layer 1	16	5	ReLU	1 × 187	16 × 183	‐	‐	Valid	1
Conv1D layer 2	16	5	ReLU	16 × 183	16× 179	‐	‐	Valid	1
MaxPool1D	‐	‐	‐	16× 179	16 × 89	‐	2	‐	1
Dropout 1	‐	‐	‐	16 × 89	16 × 89	0.1	‐	‐	‐
Conv1D layer 3	32	3	ReLU	16 × 89	32× 87	‐	‐	Valid	1
Conv1D layer 4	32	3	ReLU	32× 87	32 × 85	‐	‐	Valid	1
MaxPool1D	‐	‐	‐	32 × 85	32 × 42	‐	2	‐	1
Dropout 2	‐	‐	‐	32 × 42	32 × 42	0.1	‐	‐	‐
Conv1D layer 5	32	3	ReLU	32 × 42	32 × 40	‐	‐	Valid	1
Conv1D layer 6	32	3	ReLU	32 × 40	32 × 38	‐	‐	Valid	1
MaxPool1D	‐	‐	‐	32 × 38	32 × 19	‐	2	‐	1
Dropout 3	‐	‐	‐	32 × 19	32 × 19	0.1	‐	‐	‐
Conv1D layer 7	256	3	ReLU	32 × 19	256 × 17	‐	‐	Valid	1
Conv1D layer 8	256	3	ReLU	256 × 17	256 × 15	‐	‐	Valid	1
GlobalMaxPool1D	‐	‐	‐	256 × 15	256	‐	‐	‐	1
Dropout 4	‐	‐	‐	256	256	0.2	‐	‐	‐
Dense layer 1	64	‐	ReLU	256	64	‐	‐	‐	‐
Dense layer 2	64	‐	ReLU	64	64	‐	‐	‐	‐
Output layer	1	‐	Sigmoid	64	1	‐	‐	‐	‐

#### 2.3.7 Performance evaluation

After training the model, it is employed to test on a separate test dataset, and a range of evaluation criteria are assessed accordingly. Various critical evaluation criteria are used to measure the efficiency of the proposed method. Some of these criteria are as follows:

##### Accuracy

Accuracy, as defined by Equation 1, serves as a metric that measures the overall correctness of a model by representing the proportion of correctly identified samples in the entire dataset. Equations use True Positive (TP), False Positive (FP), True Negative (TN), and False Negative (FN) to quantify correct and mistaken identifications.

(1) $$\text{Accuracy}=\frac{\mathrm{TP}+\mathrm{TN}}{\mathrm{TP}+\mathrm{TN}+\mathrm{FP}+\mathrm{FN}}$$

Accuracy is a commonly used metric to assess the overall performance of a classification model.

##### Precision

Precision, outlined in Equation 2, functions as a gauge for the reliability of positive predictions, indicating the proportion of true positives among all samples classified as positive.

(2) $$\text{Precision}=\frac{\mathrm{TP}}{\mathrm{TP}+\mathrm{FP}}$$

##### Recall

Equation 3 defines Recall, which assesses the model's ability to detect positive instances by representing the proportion of true positives among all actual positive samples.

(3) $$\text{Recall}=\frac{\mathrm{TP}}{\mathrm{TP}+\mathrm{FN}}$$

##### F1 score

F1 Score, calculated through Equation 4, is a composite measure that balances precision and recall, providing a single value for the evaluation of the model's overall performance.

(4) $$F1\;\text{Score}=\frac{2\times \text{Precision}\times \text{Recall}}{\text{Precision}+\text{Recall}}$$

### 2.4 Additional details

In addition to the MIT‐BIH Arrhythmia examples, within the context of the PTB Diagnostic ECG dataset, our implemented CNN model, referred to as the linear deep convolutional neural network (LDCNN), serves as a valuable tool for associating nuanced waveform features with specific cardiac diseases. The model is designed to differentiate between disease types based on subtle patterns identified in ECG signals. The utilization of the PTB dataset allows our LDCNN to capture and process waveform nuances associated with various cardiac conditions, contributing to the accurate classification of different disease types. This approach enables the identification of novel markers of disease within ECG waveforms, providing insights into unique patterns that may not require processing data through the LDCNN itself. The LDCNN thus proves to be a valuable asset in detecting and understanding nuanced markers of cardiac diseases, enhancing its applicability and potential for novel disease marker discovery. However, it is important to note that our technique is invented to be adaptable and applicable to various datasets. Its strength lies in its ability to be employed across different datasets, offering a versatile solution for cardiac disease diagnosis.

### 2.5 Proposed algorithm

Algorithm 1 presents an overview of the different phases involved in our proposed method. Input to our algorithm includes the ECG signal dataset, labels corresponding to those signals, and the count of arrhythmia classes. Conversely, the algorithm yields a set of assessed performance metrics. The process begins by reading a dataset (D), addressing noise if present through Wavelet Thresholding, and standardizing features using *z*‐score normalization. If there are annotation files, beat positions and arrhythmia class information are extracted. The data are then segmented using fixed‐size window segmentation and optionally resampled. Then, the data are divided into training and test sets, and if there are more than two arrhythmia classes, one‐hot encoding is applied. The data are then transformed and fed into a convolutional neural network feature extractor, followed by training a CNN. The trained CNN is used to predict arrhythmia labels for the test set, and performance metrics are calculated and returned.

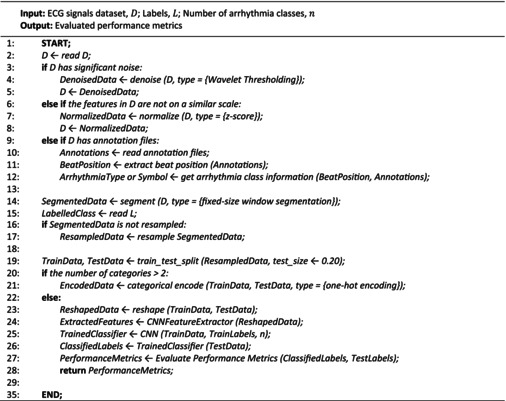

## 3 RESULTS

### 3.1 Evaluation of our proposed deep learning

**FIGURE 8:**
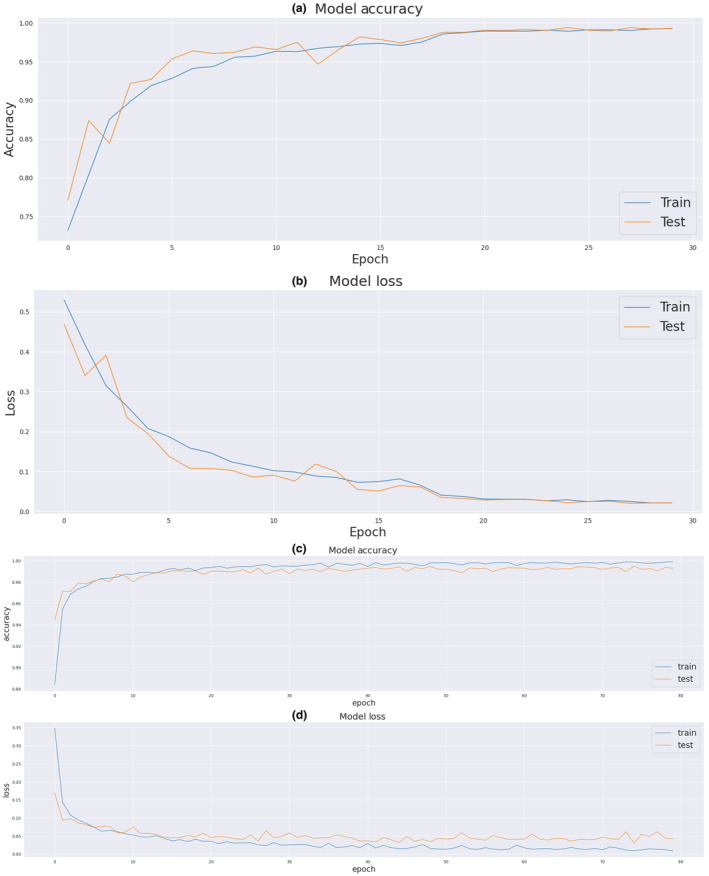
(a) Improvement in model training and testing accuracy on the PTB diagnostic ECG dataset, (b) loss trend during epochs on the PTB diagnostic ECG dataset, (c) improvement in model training and testing accuracy on the MIT‐BIH Arrhythmia dataset, and (d) loss trend across epochs on the MIT‐BIH Arrhythmia dataset.

We implemented the proposed model in Python on Google Colab as well as on a CPU with 8 GB RAM, utilizing an AMD Ryzen 55,500U processor with Radeon Graphics (running at 2.10 GHz). Figure 8 (a) illustrates the accuracy trends of model training and testing over 30 epochs on the PTB dataset. The blue curve represents changes in training set accuracy, while the orange curve represents changes in test set accuracy. Furthermore, Figure 8 (b) displays the loss trends of model training and testing across 30 epochs for the PTB dataset. The blue curve depicts changes in training set loss, while the orange curve portrays changes in test set loss. According to these results, it is possible to evaluate how much the model has improved during training and how well it maintains its performance in the test set. The accuracy of our proposed method achieves a high accuracy rate of 99.24% on this dataset.

The results of model training and testing accuracy during 80 epochs for the MIT‐BIH Arrhythmia dataset are shown in Figure 8 (c). As shown in the figure, the blue and orange curves represent the accuracy of the training set and the test set, respectively. Also, Figure 8 (d) shows the loss of model training and testing during 80 epochs for this dataset. The blue and orange curves represent the loss in the training set and the test set, respectively. As is evident, overfitting does not occur. The accuracy of our proposed method achieves a high accuracy rate of 99.38% on this dataset. In Table 10, our proposed model demonstrates robust classification performance on both the MIT‐BIH Arrhythmia and PTB Diagnostic ECG datasets, achieving high accuracy rates and class‐specific metrics. Table 11 summarizes the obtained results, showcasing impressive F1 scores, recall, precision, and overall accuracy for the proposed model across different evaluation metrics and epochs on the two datasets.

**TABLE 10:** Comparison of classification performance metrics for each class and dataset characteristics in MIT‐BIH Arrhythmia and PTB Diagnostic ECG datasets.

Datasets	Total number of samples	Training samples	Testing samples	Classes	Accuracy (%)	Precision (%)	Recall (%)	F1‐score (%)
MIT‐BIH Arrhythmia	25,000	20,000	5000	N	98.91	99.59	98.91	99.50
				L	99.90	99.80	99.90	99.80
				R	100.0	99.69	100.0	99.73
				A	98.61	99.50	98.61	99.61
				V	99.61	99.34	99.41	99.22
PTB Diagnostic ECG	14,552	11,641	2911	Normal	98.52	99.00	98.02	98.51
				Abnormal	99.62	99.24	99.62	99.43

**TABLE 11:** The obtained results of our proposed model for four evaluation metrics for two used datasets.

Method	Dataset	Epochs	Accuracy (%)	Precision (%)	Recall (%)	F1 score (%)
Proposed method	PTB	30	99.24	99.09	99.02	99.05
	MIT‐BIT	80	99.38	99.60	99.40	99.60

### 3.2 Assessment of various machine learning methods

In addition to our proposed deep learning model, we implemented and evaluated various machine learning techniques across two datasets. The results obtained for both datasets are shown in Tables 12 and 13. It can be seen in Table 12 that our proposed method performs better than the machine learning methods in the MIT‐BIH arrhythmia dataset based on F1 score, recall, precision, and accuracy. In Table 13 for the PTB Diagnostic ECG dataset, the competition between classical classification and clustering methods can be seen. These results show that our proposed method performs better than different basic machine learning algorithms according to the four evaluation indicators.

**TABLE 12:** Assessing the efficacy of our novel model against various machine learning methods using the MIT‐BIH Arrhythmia dataset.

Methods	Accuracy (%)	Precision (%)	Recall (%)	F1 score (%)
Gaussian Naive Bayes	67.0	67.4	67.0	66.8
Logistic regression	67.42	67.4	68.0	67.4
Decision trees	94.4	94.4	94.2	94.4
Linear support vector machine	94.4	94.4	94.2	94.4
Support vector machine	96.18	96.4	96.2	96.0
K nearest neighbors	97.22	97.4	97.2	97.2
Random forest	98.24	98.4	98.2	98.2
**Proposed LDCNN**	**99.38**	**99.6**	**99.4**	**99.6**

The bolding indicates that these results are particularly noteworthy and represent key findings of our study.

**TABLE 13:** Assessing the efficacy of our novel model against various machine learning methods using the PTB diagnostic ECG dataset.

Methods	Accuracy (%)	Precision (%)	Recall (%)	F1 score (%)
Gaussian Naive Bayes	61.1	65.0	68.0	60.0
Logistic regression	82.2	79.0	75.0	76.0
Linear support vector machine	82.2	79.0	75.0	77.0
Decision trees	92.1	90.0	90.0	90.5
K nearest neighbors	92.2	90.0	91.0	90.5
Support vector machine	94.48	92.0	95.0	93.0
Random forest	96.9	97.0	95.5	96.0
**Proposed LDCNN**	**99.24**	**99.09**	**99.02**	**99.05**

The bolding indicates that these results are particularly noteworthy and represent key findings of our study.

### 3.3 Comparison with different contemporary techniques

We have conducted a comparison between the proposed LDCNN method and several modern techniques in the context of arrhythmia detection. For the evaluation, we utilized the two datasets and considered all the evaluation metrics outlined in the Methods Section. According to Table 14, our proposed method has shown the best performance for all criteria compared to contemporary methods in the MIT‐BIH Arrhythmia dataset. Furthermore, Fradi et al. achieved the second‐highest performance, whereas Atal et al. exhibited the least accuracy. As indicated by the results presented in Table 15, our proposed method outperforms other contemporary techniques based on Accuracy, Recall, and F1 score, and ranks as the second‐best based on precision for the PTB Diagnostic ECG dataset. Additionally, Pham et al. secured the second‐highest ranking among performers, with Sharma et al. exhibiting the lowest level of accuracy. These analyses show that our proposed method has been validated for both the PTB and MIT‐BIH datasets as an efficient and accurate method for detecting arrhythmias in terms of various criteria.

**TABLE 14:** Comparative analysis of our innovative LDCNN technique for arrhythmia detection with a range of contemporary techniques using the MIT‐BIH Arrhythmia dataset, with metrics for all listed techniques taken directly from their respective publications.

Techniques	Accuracy (%)	Precision (%)	Recall (%)	F1 score (%)
Atal & Singh, (2020)	93.19	‐	93.98	‐
Sharma et al. (2021)	95.63	99.03	92.73	95.77
Farag, (2023)	98.18	92.44	91.90	92.17
Kumar et al. (2023)	98.66	98.92	93.88	96.34
Fradi et al. (2021)	99.34	‐	‐	99.54
**Proposed LDCNN**	**99.38**	**99.60**	**99.40**	**99.60**

The bolding indicates that these results are particularly noteworthy and represent key findings of our study.

**TABLE 15:** Comparative analysis of our innovative LDCNN technique for arrhythmia detection with a range of contemporary techniques using the PTB Diagnostic ECG dataset, with metrics for all listed techniques taken directly from their respective publications.

Techniques	Accuracy (%)	Precision (%)	Recall (%)	F1 score (%)
Sharma et al. (2021)	88.59	87.34	90.07	88.69
Wang et al. (2020)	89.87	92.86	72.38	75.23
Kumar et al. (2023)	95.79	96.29	85.38	80.37
Rafi & Ko, (2022)	98.15	97.31	96.85	97.79
Pham et al (Pham et al. (2023)	98.28	99.90	97.72	‐
**Proposed LDCNN**	**99.24**	**99.09**	**99.02**	**99.05**

The bolding indicates that these results are particularly noteworthy and represent key findings of our study.

## 4 DISCUSSION

### 4.1 Related works on cardiac arrhythmia classification from ECG signals using CNNs

In this research, we perform a comparative analysis involving five previously proposed methods from recent advances. Our goal was to evaluate and benchmark our technique against these established methods. A summary of the advantages and disadvantages of each approach is presented in Table 16.

1. Cardiac arrhythmia classification from ECG signals using a 16‐layer deep convolutional network (Yıldırım et al., 2018):
  - This approach employs a 16‐layer deep convolutional network with a 1D‐CNN architecture. This network can perform classification automatically using an end‐to‐end structure. The preprocessing of the signals involved three steps: one without normalization, another with signal rescaling within the (Singh et al., 2019) interval, and the third involving signal standardization. Finally, rescaling has achieved the best result. This network has three classification classes, which are 13, 15, and 17. The overall accuracy for each reaches 95.2%, 92.51%, and 91.33%, respectively. The highest sensitivity and specificity of 93.52% and 99.61% were achieved for 13 classes in 2018, respectively.
2. Multiple classification of heart disease using ECG signal experimental mode analysis features and one‐dimensional convolutional neural network (Hasan & Bhattacharjee, 2019):
  - In this method, a one‐dimensional CNN model mechanism is presented for the classification of arrhythmia types. The network processes the modified ECG signal as input in this context. Empirical mode decomposition methods (IMFs) and higher order intrinsic mode functions (IMFS) have been used to form the modified ECG signal. In this method, a pattern‐matching algorithm is applied using Pearson's correlation coefficient to check the ability to remove noise in the modified ECG signal. Then, the one‐dimensional CNN performs the classification operation by learning the features of the modified ECG signal. At the end of the network, the Softmax regressor activation function is used. This method has been evaluated on two MIT‐BIH ECG and arrhythmia diagnostic PTB datasets with several classes and different data registers. The results show that this method has achieved a classification accuracy of 97.70% and 98.24%, respectively, in 2019.
3. Automatic classification of arrhythmia using deep convolution neural network based on optimization (BaROA‐deep CNN) (Atal & Singh, 2020):
  - In this structure, a deep convolutional neural network is used for accurate arrhythmia classification. This network is adjusted using the BaROA optimization algorithm. The BaROA algorithm uses the integration of Bat in ROA and has two modes of encoding the solution and the fitness function. This algorithm sets the optimal weights for the deep convolutional neural network classifier, and the fitness function is used to set the weights. This method has two phases: feature extraction and arrhythmia classification. In the extraction step, the ECG signals are fed to the feature extraction module. In this module, features are extracted using wave features such as PR interval, PP interval, R peak, QT interval, and RR interval. The features are then passed to the arrhythmia classification module to classify patients as having arrhythmia or normal. The MIT‐BIH database has been used to train this network. This approach had the parameters of accuracy, sensitivity, and specificity of 93.19%, 95%, and 93.98%, respectively, in 2020.
4. Automatic diagnosis of heart disease with optimized convolutional neural networks (Fradi et al., 2021):
  - In this method, the noises of the ECG signals are removed first by preprocessing through the FIR low‐pass filter. Afterward, they enhance their dataset by extracting R–R peak information from the ECG signals. In the next step, a fully connected layer based on convolutional neural networks is trained with different optimizers. In the training process, deep learning methods and various network optimizers such as Adam (Fei et al., 2020), Nadam (Li et al., 2020), Adadelta (Qu et al., 2019), and SGD (Amari, 1993) are used. Also, to improve the performance of the neural network model, gradient optimizers are used to optimize the classification results and accuracy. Evaluation of indices for accuracy, F1‐score, sensitivity, and specificity to the values of 95%, 99%, 99.32%, and 99.63% for MIT‐BIH and to the values of 99.61%, 99%, 98.66%, and 98.85% for PTB in the year 2021 has been achieved.
5. Integrating Fuzzy Clustering and Deep Neural Networks for Heart Failure Diagnosis with ECG Data (Kumar et al., 2023):
  - This mechanism uses a framework based on deep learning and fuzzy clustering to detect arrhythmia from ECG signals. In this approach, the initial step involves preprocessing to eliminate noise from the ECG signals. Then, it uses the technique of segmenting ECG signals and balancing the classes of the dataset. The performed operations are transferred to deep convolutional neural network architecture to extract features. Finally, they employed the fuzzy clustering algorithm to classify arrhythmias, with the input being the extracted features. This approach has been trained using two MIT‐BIH and PTB Diagnostic ECG datasets, and its accuracy has reached 98% and 0.95% in both datasets, respectively, in 2023.

**TABLE 16:** Advantages and disadvantages of related works on ECG‐based cardiac arrhythmia classification using CNN.

Year	Author	Title	Algorithm	Dataset	Advantages	Disadvantages
2018	Özal Yıldırım, Paweł Pławiak, Ru‐San Tan and U. Rajendra Acharya	Arrhythmia detection using deep convolutional neural network with long‐duration ECG signals	Deep convolutional neural network	MIT‐BIH	1. Normalization. 2. Reduction of computational complexity. 3. Facilitate real‐time signal processing. 4. High accuracy of network training and validation	1. Feature extraction using end‐to‐end is not more useful for signals with high noise. 2. Not using data balancing in classes
2019	Nahian Ibn Hasan and Arnab Bhattacharjee	Deep learning approach to cardiovascular disease classification employing modified ECG signal from empirical mode decomposition	1. One‐dimensional deep convolutional neural network 2. Empirical mode decomposition (EMD) 3. Intrinsic mode functions (IMFs) 4. Stochastic gradient descent (SGD)	MIT‐BIH and PTB	1. Denoising. 2. Improving the accuracy of data validation. 3. Using learning rate scheduling to shorten training time. 4. Faster learning and reaching the highest accuracy with modified ECG	1. No adjusters (including core adjusters, bias adjusters, or activation adjusters) are used in any densely connected layer. 2. Overfitting. 3. The need for more training time to update the weights in each training step. 4. Failure to improve the proposed method with simultaneous training of IMF signals
2020	Dinesh Kumar Atal and Mukhtiar Singh	Arrhythmia classification with ECG signals based on the optimization‐enabled deep convolutional neural network	1. Optimization‐based deep convolutional neural network 2. BaROA‐based DCNN classifier algorithm	MIT‐BIH	1. More accuracy and efficiency in feature extraction. 2. Reducing the error in classification. 3. Higher convergence rate with a globally optimal solution. 3. The multi‐objective control capability of this system allows efficient arrhythmia classification	1. This method may require complex and expensive equipment when using sensors. 2. The use of the multiresolution wavelet‐based method can lead to higher computational complexity and increased demand for computing resources. 3. It has a higher tendency to avoid local optima mechanisms
2021	Marwa Fradi, Lazhar Khriji, Mohsen Machhout and Abdulnasir Hossen	Automatic heart disease class detection using convolutional neural network architecture‐based various optimizers‐networks	1. Convolutional neural network 2. Finite impulse response (FIR) filter	MIT‐BIH and PTB	1. Denoising. 2. Short processing time. 3. Using optimizers and improving cost performance. 4. Low computational complexity. 5. Increasing the learning rate and convergence speed	1. The implementation of a CNN architecture using a GPU can be limited by hardware and software resources. 2. Technical complications in implementing algorithms on GPU
2023	Sanjay Kumar, Abhishek Mallik, Akshi Kumar, Javier Del Ser and Guang Yang	Fuzz‐ClustNet: Coupled fuzzy clustering and deep neural networks for arrhythmia detection from ECG signals	1. Deep convolutional neural network 2. Fuzzy clustering algorithm	MIT‐BIH and PTB	1. Denoising. 2. Segmentation of the signals. 2. Balance the classes by augmentation. 3. Optimal feature extraction. 4. Increasing the accuracy of diagnosis	1. Absence of the 3/2 to 1/3 rule in training and testing. 2. Not using validation data to prevent overfitting in the training phase. 3. Computational complexity

In response to RQ1, our linear deep convolutional neural network (LDCNN) achieves remarkable diagnostic effectiveness, boasting 99.24% accuracy on PTB and 99.38% on MIT‐BIH datasets. Addressing RQ2, the LDCNN consistently outperforms traditional methods, surpassing them in F1 scores, precision, recall, and overall accuracy. RQ3 findings reveal the LDCNN's consistent diagnostic accuracy across arrhythmia classes. Finally, RQ4 highlights stable training trends, absence of overfitting, and effective convergence, affirming the LDCNN's adaptability and robust learning, ultimately contributing to its efficacy in cardiac disease diagnosis across diverse datasets.

In our simulation on the MIT‐BIH and PTB datasets, we achieved remarkable maximum accuracies of 99.38% and 99.24%, respectively. Our approach involves processing digital data directly, converting it into vectors, and utilizing datasets as digital numbers. Despite the one‐dimensional nature of the data, our method adeptly addresses denoising and other issues, automatically identifying patterns for arrhythmia detection. The high accuracy underscores the success of our architecture in solving the problem without the need for analog‐to‐digital conversion or sampling.

The strength of our method lies in its direct approach to digital data processing, eliminating errors associated with sampling. The proposed LDCNN method effectively covers potential errors related to sampling, ensuring signal quality and accuracy without the need for additional conversion steps. This result demonstrates the correctness and efficacy of our approach.

### 4.2 Future works

According to the obtained results and considering the observed needs, future research could explore the integration of additional characteristics such as blood pressure and cholesterol levels with arrhythmia signals. Additionally, developing an Internet of Things (IoT) device to predict and detect arrhythmias in real‐time, and employing artificial intelligence for long‐term ECG data analysis, could significantly enhance personalized treatment plans. Ensuring data security through blockchain technology could also be a valuable approach to protect patient privacy while allowing for broader analysis of anonymized data.

## FUNDING INFORMATION

This research was conducted without external funding or institutional support.

## CONFLICT OF INTEREST STATEMENT

The authors declare that they have no conflicts of interest to disclose.

## ETHICS STATEMENT

This research utilizes the publicly available PTB Diagnostic ECG and MIT‐BIH Arrhythmia datasets for ECG signal analysis in cardiac disease diagnosis. The study adheres to relevant regulations and the ethical guidelines of Physiological Reports. No direct human interactions were involved.

## CODE AVAILABILITY

The code, including the deep learning and machine learning models, preprocessing scripts, and architectures with Python, used in this study, is available at the following GitHub repository: https://github.com/aliebayani/Linear‐Deep‐Convolutional‐Neural‐Network‐LDCNN.git.

## Data Availability

Dataset 1 (MIT‐BIH Arrhythmia dataset): The MIT‐BIH Arrhythmia dataset used in this research is available at the following DOI: https://doi.org/10.13026/C2F305. You can access this dataset by visiting the provided DOI. Dataset 2 (PTB Diagnostic ECG dataset): The PTB Diagnostic ECG dataset is accessible through the following DOI: https://doi.org/10.13026/C28C71. Additionally, the annotated PTB dataset is available on Kaggle at https://www.kaggle.com/datasets/shayanfazeli/heartbeat.
